# The Effects of Working Hours on Nerve Conduction Test in Computer Operators

**DOI:** 10.5704/MOJ.1303.008

**Published:** 2013-03

**Authors:** AA Ganeriwal, DA Biswas, TK Srivastava

**Affiliations:** Department of Physiology, Jawaharlal Nehru Medical College, Sawangi (Meghe) Wardha, India; Department of Physiology, Jawaharlal Nehru Medical College, Sawangi (Meghe) Wardha, India; Department of Physiology, Jawaharlal Nehru Medical College, Sawangi (Meghe) Wardha, India

## Abstract

Background:Long hours of work at a computer can result in potential adverse effects such as pain, paraesthesia, and subjective weakness of upper extremities with associated decreased nerve conduction velocities of peripheral nerves (median and ulnar). Objective: To determine whether repetitive tasks performed for long hours by computer operators can lead to peripheral neuropathy as measured by nerve conduction studies of upper extremities. Material And Methods: We performed nerve conduction tests on 50 computer operators in two study groups. Group I consisted of computer operators who worked a minimum of 6 hours per day at the computer while Group II worked a maximum of 2 hours per day. Results: Significant differences were observed between groups for most physical signs. In addition, there were significant differences between the groups for nerve conduction velocities of the medial (motor & sensory) and ulnar (motor & sensory) nerves. Conclusion: We found symptoms of pain, paraesthesia and subjective weaknesses as well as decreased conduction velocities of peripheral nerves in computer operators who work long hours. Further nerve conduction studies are needed to confirm predictive value for the development of carpal tunnel syndrome.

**Key Words:**

computer operator, long work hours, nerve conduction studies

## Introduction

The information technology (IT) revolution has embraced everyone in its stride. People are on the verge of becoming computer savvy or already are computer literate. As a coin has two sides, the IT revolution has proved to be simultaneously a boon and a bane. It is a boon as it facilitates data interpretation, faster communication, speeds work completion and much more; on the other hand, it is a bane for those involved in providing the IT sector with its true face value. Here, we mean the millions of computer users across the globe (~77 million people as of October 2003 according o the Bureau of Labor Statistics, US Department of Labor)^1^. Many studies have sought to ascertain what leads to upper limb disorders in computer operators and several hypotheses have been put forth, but none dissects the exact cause. Certainly, we saw that increased computer work coincided with a higher prevalence of work-related musculoskeletal disorders of the upper extremities (WRMSDs)^2^. These WRMSDs are defined as injuries or disorders of the muscles, nerves, tendons, joints, cartilage, and spinal discs associated with exposure to risk factors in the workplace^3^. WRMSDs of the hand and wrist are associated with the longest absence from work and are therefore, associated with greater decrease in productivity and wages lost. Thus, employees are affected by functional impairment and lost wages while employers are affected by decreased productivity and increased disability payments.

The characteristic features of WRMSD are pain, associated paraesthesia, subjective weakness and forearm weakness and fatigue with regular computer use. It is therefore important to consider peripheral nerve involvement in the presence of this triad of symptoms (mainly pain, paraesthesia, and subjective weakness)^4^. Progressive functional impairments develop with chronic repetitive tasks and accompany signs of tissue injury. Studies of median nerve function among computer users revealed exposure characteristics in computer workers; median nerve function has also been evaluated for use in diagnosis and possible pathophysiological mechanisms^5^.

Results of various epidemiological studies investigating computer usage hours and WRMSD have not been consistent. Accurate identification of factors that predict chronic disability may also shed some light on why computer workers develop disability and thus guide the development of interventional strategies for prevention of these injuries. Precise and accurate diagnosis is crucial for effective management and rehabilitation and also to enable epidemiological investigators to establish causation.

The aim of this study was to determine the effect of wrist posture on nerve conduction studies in computer operators. Our objectives were to study nerve conduction velocity of the median and ulnar nerves in the mouse-operating limb and to investigate WRMSD in computer users in relation to hours of computer use per day.

## Materials and Methods

The study was conducted in the Neurophysiology laboratory of the Department of Physiology , Jawaharlal Nehru Medical College, Sawangi (Meghe), Wardha. The sample included 50 computer operators: group I, with 25 right handed office workers who worked at a computer a minimum of 6 hours per day on the college campus; and group II, with 25 right handed individuals who worked at computer for 2 hours or less per day. Study participants in the two groups were age matched (i.e., 20-40 years of age). Computer operators of both sexes were included in this study.

Exclusion criteria consisted of the following: relevant history of a fall, accidents or other disease pertaining to soft tissue injury or pain (i.e., rheumatoid arthritis, diabetes, and anaemia); and persons who were alcoholics.

The institutional ethical committee of Datte Meghe Institute of Medical Sciences approved this study. Written informed consent was obtained from all participants before study participation.

Anthropometric measures were obtained and BMI was calculated. Vital signs were also recorded and pain was evaluated using a visual analogue scale (VAS scale, 0-9)^4^.

A questionnaire was developed to ascertain the duration of computer working hours, any associated pain, location of pain, (wrist, elbow, shoulder, neck)^2^, any sensation of tingling numbness and subjective fatigability/weakness, in the mousing hand (right hand). The questionnaire was designed in the local language, to facilitate understanding of the questionnaire material. Subjects were also queried to determine if they received any treatment for any of their ailments.

Next, study participants underwent nerve conduction studies including electrophysiological measures (RMS EMG EP Mark II Recorders and Medicare Systems, Chandigarh India), using established methodology described by Mishra and Kalita^6^.

Patients relaxed on a couch in a soundproof and air- conditioned examination room to avoid muscle artefacts. Studies included conduction velocity for motor nerve conduction and sensory nerve testing on the median and ulnar nerves.

## Discussion and Results

WRMSD are injuries and disorders of the nerves, muscles and tendons are associated with repeated exposure to various risk factors. The use of computers involves continuous repetitive strokes, persistent wrist extension during typing, and complete pronation of the forearm for operation of the computer mouse. All of these tasks involve repetitive motions, rendering computer operators more susceptible to WRMSD.

Complications associated with computer use are usually manifested as upper limb disorders namely pain (wrist, elbow, shoulder and neck), tingling, numbness, fatigue and paraesthesias. These symptoms are typically found in computer operators who work long work hours at their computers. At the onset of such symptoms, computer operators should undergo detailed clinical and neurological evaluation including nerve conduction tests.

Previously reported prevalence of the following symptoms ranges from 0.7% to 34.8%: self-reported musculoskeletal discomfort (MSD) symptoms such as fatigue, hand or wrist pain, backache, headache, leg cramps, leg stiffness, numbness in ankles and feet, reduction in hand strength and difficulty grasping objects, neck and shoulder stiffness, neck and shoulder pain or tingling/numbness in hands or fingers during or after work or at night perhaps interrupting sleep^7^.

In the present study, the two groups were age, height and weight matched with no significant difference in anthropometric data or calculated BMI (Table I). Werner et al.^8^ reported that obese (BMI > 29) individuals were four times more prone to present with median mononeuropathy than workers who were normal weight or slender (BMI < 25) and a cross-sectional and longitudinal study by Nathan et al.^9^ involving 429 workers showed that obesity is a risk factor for slowing of sensory conduction of the median nerve in industry workers. In addition, age is an independent risk factor that influences the prevalence of median mononeuropathies among active workers.

**Fig. 1:**
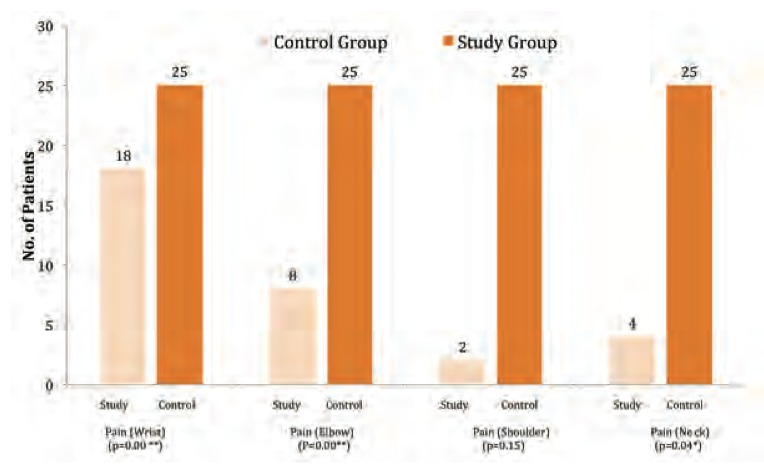
: Comparison of the physical signs of pain at the wrist, elbow, shoulder and neck. Study group, computer operators, minimum of 6 hours /day; control group, computer operators, maximum of 2 hours/day ** highly significant

Consistent with the work of Jepsen and Thomsen^4^, we found significant differences in reported pain between the two study groups except for the shoulder region (p<0.01; Figure 1). In the Jepsen study, upper limb pain in computer operators similar in character to neuropathic pain was evoked spontaneously or as an abnormal response to stimuli, and allodynia occurred frequently.

Palmer et al. found increased pain in the neck and shoulder region while we did not find a significant difference in shoulder pain between the two groups (p value>0.05)^10^. Such pain may result from continuous friction caused to the nerve in the limited available space near the joints.

**Fig. 2:**
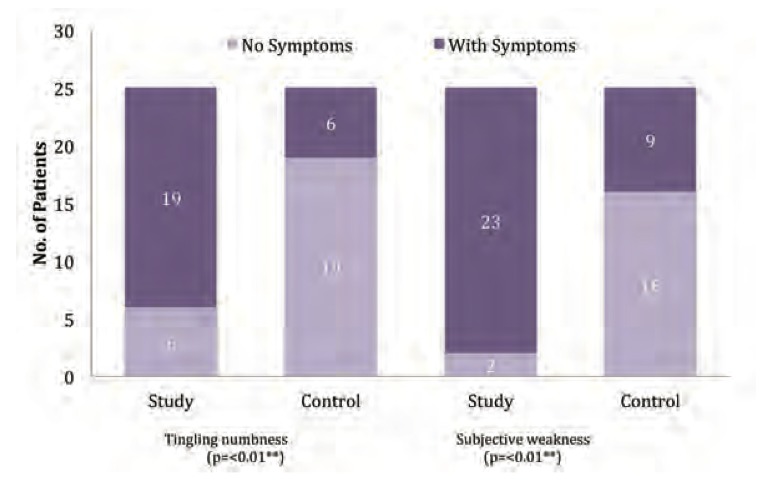
: Comparison of tingling numbness and subjective weakness. Study group, computer operators, minimum of 6 hours /day; control group, computer operators, maximum of 2 hours/day ** highly significant

We found a significant difference between the two groups for subjective weakness (p value < 0.01; Figure 2). This is due to the muscular imbalance in the mouse-operating limb as some muscles are progressively shortened and the associated antagonists are then passively stretched. Our findings are consistent with that of Thomsen et al. who found that individuals with moderate to severe forearm pain had a decreased forearm extensor muscle fatigue response^11^. This weakness may further deteriorate with the passage of time and increased computer usage. Pain caused by repeated friction and tension, leads to inflammatory changes and perineural oedema, which in turn hampers the normal nerve function and axoplasmic transport. This leads to fatigue due to disruption of nutrient transport to the nerve and decreased vascular perfusion. The Thomsen group hypothesized the pathophysiology of upper extremity muscle disorders including forearm pain in computer users is caused by disordered muscle cells or impaired local circulation^11^.

Repetitive stressful movements involving peripheral nerves may lead to nerve entrapment, which is often associated with tingling and/or numbness without pain found in many of computer users. As expected, we found a significant difference between the two groups for this symptom (p value < 0.01; Figure 2). Our findings are consistent with those reported by Anderson et al. showing that tingling or numbness in the right hand was associated with time spent using a mouse and prevalence of tingling/numbness in the median nerve of 4.8%12. Further, Overgaard et al. demonstrated that tingling/numbness of the hands and fingers among computer users is an early sign of nerve compression^13^.

Our findings of significant differences in pain, tingling numbness and subjective weakness between the two groups were similar to those reported by Jepsen and Thomsen; these authors suggested that peripheral nerve-involvement should be suspected in the presence of the common presence of the symptom triad of pain, paraesthesia and subjective weakness, and from physical findings suggesting neuropathy^4^.

**Fig. 3:**
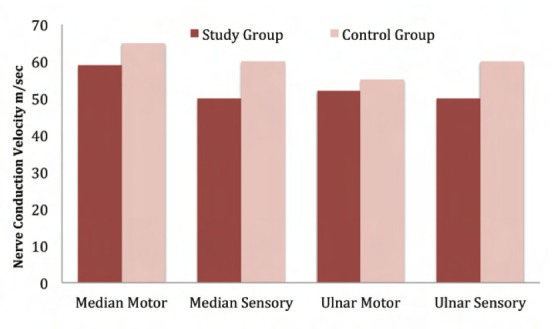
: Nerve conduction velocity of median (motor & sensory) and ulnar (motor & sensory) nerves. Study group, computer operators, minimum of 6 hours/ day; control group, computer operators, maximum of 2 hours/ day.

Table II shows that conduction velocity is decreased in the median motor nerve, the median sensory nerve, the ulnar motor nerve and the ulnar sensory nerve in group I compared to group II. Independent t-test analysis showed this difference to be highly significant (p value<0.01; Figure 3). These findings were consistent with those of Murata et al. who assessed employees entering data for more than 6 hrs./day, and found significant differences in median sensory conduction velocities^14^. In contrast, Sanden et al. reported no statistically significant difference in median nerve conduction velocity^15^. Due to the repetitive movements inherent in computer use, friction is produced, which may lead to the inflammatory changes and resultant nerve compression. This compression leads to mechanical disruption of the blood nerve barrier, and indirectly compromises neural function by restricting vascular perfusion. Increased intraneural pressure consequently leads to oedema, which further compresses the nerves especially at joints and bony prominences. Depending upon the magnitude and duration of nerve loading from prolonged computer working hours, increased localized pressure leads to axonal and myelin sheath disruption. This, in turn, increases the distances between the nodes of Ranvier and interferes with impulse transmission thus decreasing conduction velocity. At times, this compression causes axonal demyelination and degeneration, and when chronically present may induce neural fibrosis.

The central nervous system can be profoundly affected by performance of repetitive and intensive hand movements. Chronic pain, inflammation and peripheral nerve tissue injury result in chronic overstimulation of nociceptive afferents terminating in the spinal dorsal horns^3^. Although these inflammatory processes may resolve and scarring of the tissues may subside with accompanying healing process, if the initial stimulus of the continual exposure is still persistent, chronic inflammation can result in a chronic fibrotic state.

**Fig. 4:**
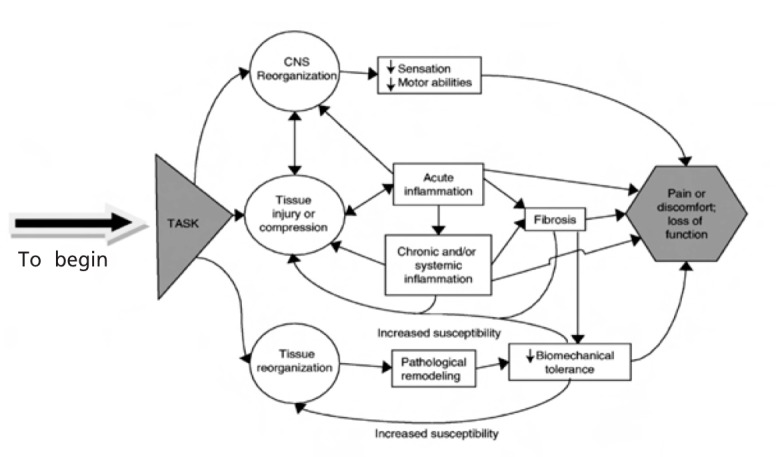
: Schematic diagram showing the 3 primary pathways hypothesized to lead to work-related musculoskeletal disorders caused by repetitive and/or forceful hand-intensive tasks. adapted from Work-Related Musculoskeletal Disorders of the Hand and Wrist:

The presence of perineural oedema and neural compression at the wrist may reflect an increase in carpal tunnel pressure.Carpel tunnel pressure can increase in the presence of persistent continuous wrist flexion and extension. It is thus likely that these repetitive movements lead to compression of the median nerve at the wrist. In recent years, with the advent of increasing computer use, a question arises as to whether excessive computer usage leads to carpal tunnel syndrome and if so, should this be considered an occupational hazard (Figure 4).

Continuing the discussion, the following studies report on the development of work related musculoskeletal disorders in computer operators. Gerr et al. studied hand and arm WRMSD common among computer users and reported that more than 50% of these users reported symptoms within 1 year of starting a new job and 64% to 73% of those who reported symptoms had confirmed WRMSD diagnosis. Hagberg et al. reported prevalent upper extremity WRMSD among computer users with a resulting impact on productivity. In contrast, Stevens et al. showed that the frequency of carpal tunnel syndromes among computer users was no different from the general population 3. The question of whether these these nerve conduction studies have predictive value for future development of carpal tunnel syndrome was brought by Nathan et al^16^. Another study done by Myers 17 showed that among individual hands, nerve conduction abnormalities tended persist to persist (82% had 11-year persistence), despite wide fluctuation of symptoms (13% had 11-year persistence). There was a strong, direct linear correlation between initial severity of symptoms and slowing and subsequent development of carpal tunnel syndrome in the Meyer study.

**Table I:**
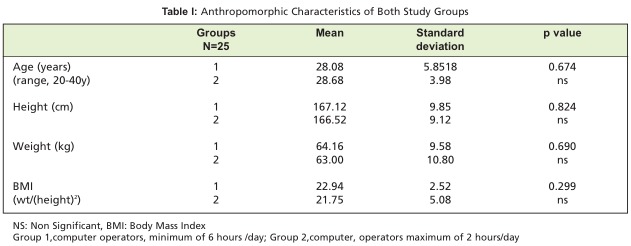
: Anthropomorphic Characteristics of Both Study Groups

**Table II:**
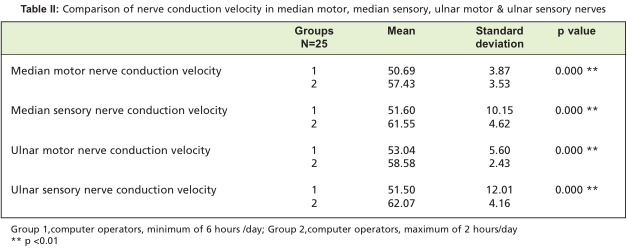
: Comparison of nerve conduction velocity in median motor, median sensory, ulnar motor & ulnar sensory nerves

## Conclusion

Hand and wrist work-related musculoskeletal disorders comprise a significant fraction of substantial work related illness and are associated with high medical costs and loss of workplace productivity. As expected, we observed that computer operators who work long hours at the computer are at risk for repetitive stress injury with symptoms such as pain, tingling numbness, weakness and associated neural involvement in the mouse operating limbs. Decreased conduction velocities confirm peripheral neural involvement (median and ulnar nerves) in these individuals. Studies have shown that there is injury, circulatory insufficiency and central nervous system involvement that lead to development of peripheral neuropathy in computer operators who work long hours at the computer. Further studies are required to determine if nerve conduction study results are predictive of carpal tunnel syndrome development. A larger sample size is required to shed light on the prevalence of this problem in India.
